# Supplementary material for: Near-infrared-laser-navigated dancing bubble within water via a thermally conductive interface
Source: Nat Commun. 2022 Sep 30;13:5749. doi: 10.1038/s41467-022-33424-4 (PMC9525293; doi:10.1038/s41467-022-33424-4)
Supplement: Supplementary file 1 — Supplementary Information [file 41467_2022_33424_MOESM1_ESM.pdf]

Supplementary Materials for Near-infrared-laser-navigated dancing bubble within water via a thermally conductive interface

Man Hu[†*], Feng Wang[†], Li Chen, Peng Huo, Yuqi Li, Xi Gu, Kai Leong Chong, Daosheng Deng[*]
[*]Corresponding author. E-mail: human@fudan.edu.cn; dsdeng@fudan.edu.cn
[†]These authors contributed equally to this work.

This PDF file includes:

Supplementary Notes 1 to 8

Supplementary Methods

Supplementary Fig. 1 to 13

Supplementary Tab. 1 to 4

Supplementary References

Other Supplementary Materials for this manuscript include the following:

Supplementary Movies 1 to 10

Supplementary Notes

Supplementary Note 1. Reproducibility of bouncing behavior

The data for the periodic bouncing are well reproducible in experiments for three times at three different laser power ($P = 15, 20, 25$ W), as shown in Supplementary Fig. 2. The topmost position of the bubble H_{t} (as depicted in Fig. 1a in the main text, the vertical distance from the topmost of the bubble to the solid cover) is presented in Supplementary Fig. 2b. The periodical displacement of the bubble implies the occurrence of the bouncing behavior, including the bouncing onset and stop moment, and the bouncing amplitude.

Supplementary Note 2. The effect of cover materials on the bouncing behavior

To confirm the effect of high thermal conductivity of the top glass on bubble behaviors, we carried out experiments for different cover materials with various thermal conductivity (see Supplementary Tab. 1). For the higher thermal conductivity such as quartz and sapphire cover, the bubble bouncing behavior is observed, but bouncing is absent for the lower thermal conductivity such as PMMA and PDMS cover.

The snapshots shown in Supplementary Fig. 3a demonstrate the bubble dynamics without bouncing for a PMMA cover with a low thermal conductivity of 0.19 W/(m·K). For PMMA and sapphire cover, the bubble radius R, center position H_{c}, and topmost position H_{t} are compared in Supplementary Fig. 3b-d respectively. For a PMMA cover, no bounce is observed, and the bubble is just hanging at the solid-liquid interface.

Supplementary Note 3. Observation of peak temperature and temperature inversion layer (TIL)

T_{peak} and the corresponding thickness δ_{inv} are shown in Supplementary Fig. 4a for $P = 15$ W with bubble formation. δ_{inv} remains almost constant about 0.3 mm during laser irradiation. During the pre-heating stage within 1 s upon laser impacting on water, the elevated temperature ($\Delta T = T_{\text{peak}} - T_0$, T_0 for room temperature) nearly increases linearly with heating time ($t_{\text{laser on}}$) for $P = 15$ W with bubble formation (Supplementary Fig. 4b) and $P = 10$ W without bubble formation until it reaches the characteristic timescale for heat transfer τ_{c} (Supplementary Fig. 4c).

For $P = 15$ W with bubble formation, from the temperature profile along the Z direction in Supplementary Fig. 4d, we obtain the magnitude of the temperature gradient $\mathrm{d}T/\mathrm{d}Z \approx 50$ K/mm (Supplementary Fig. 4e), which is strong enough to induce downward thermal Marangoni force against buoyancy force.

Supplementary Note 4. Model for peak temperature evolution

To model the temperature evolution, the laser is treated as a collimated laser beam with Gaussian distribution along the r direction, and the related parameters are the radius $r_{\text{l}} = 0.5$ mm, average laser power $P = 15$ W, and penetration depth $\delta = 2.2$ cm. The laser intensity distribution is,

$$I\left(r\right) = \frac{P}{\pi r_{\text{l}}^2} e^{-r^2/r_{\text{l}}^2} \tag{1}$$

The deposited local energy intensity is obtained from Beer-Lambert law in Ref. (*1*):

$$G\left(r, z\right) = \frac{\alpha \eta \left(1 - R_{\text{c}}\right) P}{\pi r_{\text{l}}^2} e^{-r^2/r_{\text{l}}^2} e^{-\alpha z} = A_0 P \cdot e^{-r^2/r_{\text{l}}^2} e^{-\alpha z} \tag{2}$$

where $\alpha = 1/\delta$ is the attenuation constant, R_{c} is the reflection coefficient, and η is a constant coefficient for heating water by considering the latent heat effect. The deposited laser

energy mainly causes the heating and vaporization of water, $P\tau = c_{\mathrm{p}} m_{\mathrm{h}} \Delta T + L_{\mathrm{v}} m_{\mathrm{v}}$, where $c_{\mathrm{p}} = 4.2$ kJ/(kg· K) is the heat capacity, $L_{\mathrm{v}} = 2257.2$ kJ/kg is the latent heat of water for vaporization, $m_{\mathrm{h}}, m_{\mathrm{v}}$ are the mass of heated water and vaporized water. Thus, the fraction of the deposited laser energy for heating can be estimated $\eta \approx c_{\mathrm{p}}\Delta T/(c_{\mathrm{p}}\Delta T + L_{\mathrm{v}}) = 0.13$. $A_0 = \alpha\eta\left(1 - R_{\mathrm{c}}\right)/\pi r_{\mathrm{l}}^2$ is an absorption constant related with laser parameters.

For temperature evolution within a short time, by neglecting convection and diffusion along the r direction, the model is simplified to 1D heat conduction equation,

$$\frac{\partial T}{\partial \tau} = \kappa \frac{\partial^2 T}{\partial z^2} + \frac{A_0 P}{\rho c_{\mathrm{p}}} e^{-r^2/r_{\mathrm{l}}^2} e^{-\alpha z} \tag{3}$$

where $\kappa = k/\rho c_{\mathrm{p}}$ is thermal diffusivity of water, k is thermal conductivity.

By further ignoring diffusion along z direction, a trivial solution is obtained,

$$T\left(r, z, \tau\right) - T_0 = \frac{A_0 P}{\rho c_{\mathrm{p}}} e^{-r^2/r_{\mathrm{l}}^2} e^{-\alpha z} \tau \propto P\tau. \tag{4}$$

which is consistent with the experimental observation ($\Delta T \propto t_{\mathrm{laser\ on}}$) in Supplementary Fig. 4b and Supplementary Fig. 4c.

For temperature evolution during the long time period, by neglecting convection and considering the thermal diffusion along the r direction, the steady-state axisymmetric heat conduction equation is,

$$\frac{1}{r}\frac{\partial}{\partial r}\left(r\frac{\partial T}{\partial r}\right) + \frac{A_0 P}{\kappa} e^{-r^2/r_{\mathrm{l}}^2} = 0 \tag{5}$$

The boundary conditions are as below,

$$\left.\frac{\mathrm{d}T}{\mathrm{d}r}\right|_{r=0} = 0, \tag{6}$$

$$T(r_{\mathrm{w}}) = T_0. \tag{7}$$

Here the first boundary condition (Eq. 6) results from the symmetry, and the second condition (Eq. 7) is a constant temperature for the far field condition, where r_{w} is the width of water bulk ($r_{\mathrm{w}} = 5$ mm in experiments).

For sufficiently long time period, the diffusion length of heat transfer is much larger than the beam size of laser spot, and the laser intensity profile (the term $e^{-r^2/r_{\mathrm{l}}^2}$) is simplified to a point source of heat. Then, according to Ref. (*2*), the analytical solution is,

$$\Delta T = T(r) - T_0 = \frac{A_0 P r_{\mathrm{w}}^2}{4\kappa}\left(1 - \frac{r^2}{r_{\mathrm{w}}^2}\right) \tag{8}$$

which reaches a constant temperature for long time period, thus transition regime is observed for intermediate time period as shown in Supplementary Fig. 4b and Supplementary Fig. 4c.

Supplementary Note 5. Model for velocity and thermal boundary layer

To model the boundary layer induced by the buoyancy flow sweeping the cover surface, we consider the buoyancy flow similar to the analysis for laminar natural convection on a heated vertical surface in Ref. (*2*). The sketch is shown in Fig.2c in the main text, and the governing equation with Boussinesq approximation is as below,

$$u\frac{\partial u}{\partial z} + v\frac{\partial u}{\partial r} = g\beta\left(T - T_0\right) + \frac{\mu}{\rho}\frac{\partial^2 u}{\partial r^2}, \tag{9}$$

where β is thermal expansion coefficient of water. Then, we choose the beam size as characteristic length scale $L = r_{\mathrm{l}}$, and the buoyancy flow velocity V_{b} as the characteristic velocity to dimensionless Eq. 9. Introducing $r^* = r/L, z^* = z/L, u^* = u/V_{\mathrm{b}}, v^* = v/V_{\mathrm{b}}, T^* = (T - T_0)/(T_{\mathrm{m}} - T_0)$, the dimensionless governing equation is obtained as below,

$$u^*\frac{\partial u^*}{\partial z^*} + v^*\frac{\partial u^*}{\partial r^*} = \frac{g\beta\left(T_{\mathrm{m}} - T_0\right)L}{V_{\mathrm{b}}^2}T^* + \frac{1}{\mathrm{Re_L}}\frac{\partial^2 u^*}{\partial r^{*2}}. \tag{10}$$

Here $V_{\mathrm{b}} = \sqrt{g\beta\left(T_{\mathrm{m}} - T_0\right)L}$, $\mathrm{Re_L} = \rho V_{\mathrm{b}} L/\mu = \mathrm{Gr}^{1/2}$, where Grashof number $\mathrm{Gr} = \rho^2 g\beta\left(T_{\mathrm{m}} - T_0\right)L^3/\mu^2$ is the ratio of the buoyancy forces to the viscous forces.

To model the buoyancy flow sweeping the cover surface, the hydrodynamic solution by Blasius for laminar flow over isothermal plate in Ref. (*2*) is applied, and thickness of velocity boundary layer (δ_{v}) is attained,

$$\delta_{\mathrm{v}} = \frac{5L}{\sqrt{\mathrm{Re}}} = 5\sqrt{\frac{\mu L}{\rho V_{\mathrm{b}}}} = 5\sqrt{\frac{\mu L}{\rho\sqrt{g\beta\left(T_{\mathrm{m}} - T_0\right)L}}} \propto \Delta T^{-1/4} \tag{11}$$

This scaling relationship $\delta_{\mathrm{v}} \propto \Delta T^{-1/4} \propto P^{-1/4}$ is obtained according to Eq. 4 and Eq. 8.

The thickness of thermal boundary layer thickness according to Ref. (*2*) for $\mathrm{Pr} \geq 0.6$ ($\mathrm{Pr} = c_{\mathrm{p}}\mu/k \approx 7$ for water at 293 K) is as following,

$$\delta_{\mathrm{th}} = \frac{\delta_{\mathrm{v}}}{\mathrm{Pr}^{1/3}} \propto P^{-1/4}. \tag{12}$$

Within the thermal boundary layer, thermal diffusion is dominant, while outside the thermal boundary layer, thermal convection is dominant.

According to previous analysis, the velocity of buoyancy flow is $V_{\mathrm{b}} = \sqrt{g\beta\left(T_{\mathrm{m}} - T_0\right)L}$. By setting the temperature difference as the difference between boiling point ($T_{\mathrm{m}} = 373$ K) and initial temperature of water ($T_0 = 293$ K) and the radius of laser beam as typical length scale $L = 0.5$ mm, the estimated value of buoyancy flow velocity is $V_{\mathrm{b}} \approx 8.9$ mm/s.

To visualize the thermal buoyancy flow induced by laser irradiation in experiments, PIV method is applied. Polystyrene (PS) particles (purchased from KBSpheres, diameter $d = 40\mu$m) with particle concentration c =2.5 mg/mL were dispersed into an aqueous glycerol solution with 33% glycerol by volume in order to match the density of PS particles. The flow field (Supplementary Fig. 5) is comparable with simulation, and the maximum upward velocity about 10 mm/s also agrees well with the theoretical estimation.

Supplementary Note 6. Model for temperature inversion layer

To understand TIL qualitatively, we use the model solution for interfacial contact between two semi-infinite solids at different initial temperatures in Ref. (*3*). Although the thickness of cover and boundary layer is finite, the model may qualitatively demonstrate the relevant physical parameters for TIL.

By assuming the sapphire glass with an initial temperature T_0 contacts the heated water with temperature $T(0, z, t)$, the temperature distribution in water has an analytical solution in Ref. (*3*),

$$\hat{T}(0, z, t) = T_0 + \frac{T(0, z, t) - T_0}{1 + e_\mathrm{s}/e_\mathrm{w}} \left[1 + \frac{e_\mathrm{s}}{e_\mathrm{w}} \mathrm{erf}\left(\frac{z}{\eta}\right)\right], \qquad (13)$$

where $e_\mathrm{i} = \sqrt{k_\mathrm{i} \rho_\mathrm{i} c_\mathrm{pi}}$ is thermal effusivity of material, i=s,w for solid cover (sapphire) and water. $\eta = \min(\delta_\mathrm{d}, \delta_\mathrm{th})$ is the minimum between the thermal diffusion length ($\delta_\mathrm{d} = 2\sqrt{\kappa t}$) and thermal boundary layer thickness (δ_th), indicating that the maximum thermal diffusion length is limited by thermal boundary layer.

The temperature distribution for cover materials with different thermal conductivity (cover thickness $e = 0.25$ mm) is demonstrated in Supplementary Fig. 6a, indicating T_peak arises from the higher thermal conductivity of the quartz and sapphire glass. The green line in Supplementary Fig. 6a also indicates that the temperature gradient within TIL is too weak for the PMMA cover to drive bubble bouncing. The temperature distribution for sapphire cover with different thickness (e) is demonstrated in Supplementary Fig. 6b, indicating temperature gradient is dependent on cover thickness.

To determine the thickness of TIL δ_inv, let the derivative of $\hat{T}$ equal zero for a given time t_0 in short time period for $\eta = \delta_\mathrm{th}$,

$$\frac{\partial \hat{T}(0, z, t_0)}{\partial z} = \frac{A_0 P t_0 e^{-\alpha z}}{\rho c_\mathrm{p}(1 + e_\mathrm{s}/e_\mathrm{w})} \left[\frac{e_\mathrm{s}}{e_\mathrm{w}} \cdot \frac{2}{\delta_\mathrm{th}\sqrt{\pi}} e^{-z^2/\delta_\mathrm{th}^2} - \alpha \left(1 + \frac{e_\mathrm{s}}{e_\mathrm{w}} \mathrm{erf}\left(\frac{z}{\delta_\mathrm{th}}\right)\right)\right] = 0. \qquad (14)$$

Then, the thickness of TIL can be obtained by solving

$$\frac{2}{\delta_{\text{th}}\sqrt{\pi}}e^{-z^2/\delta_{\text{th}}^2} - \alpha\left(\frac{e_{\text{w}}}{e_{\text{s}}} + \text{erf}\left(\frac{z}{\delta_{\text{th}}}\right)\right) = 0. \tag{15}$$

Qualitatively, the thickness of TIL δ_{inv} is related with not only the thickness of thermal boundary layer (δ_{th}) corresponding to the physical properties and flow status of liquid, about also the thermal effusivity corresponding to the thermal properties of cover materials. The thickness of TIL (δ_{inv}) increases with the thermal effusivity of cover material, as shown in Supplementary Fig. 6c.

For the analysis in our experiments, because of the large thermal effusivity of sapphire, the thickness of TIL (δ_{inv}) can be reasonably assumed to be equal with the thickness of thermal boundary layer for the simplicity, $\delta_{\text{inv}} \approx \delta_{\text{th}}$.

Additionally, the thickness of TIL δ_{inv} at different laser power P is extracted from both experiments and simulation by COMSOL (the thickness of sapphire cover e =250 μm). This layer thickness dependent on the laser power (P) (Supplementary Fig. 6d) agrees well with the $-1/4$ scaling law for thermal boundary layer (Eq. 11 and Eq. 12),

$$\delta_{\text{inv}} \approx \delta_{\text{th}} \propto P^{-1/4}. \tag{16}$$

Moreover, according to previous analysis, the thickness of thermal boundary layer $\delta_{\text{th}} = \frac{\delta_{\text{v}}}{\text{Pr}^{1/3}} = \frac{5L}{\text{Re}^{1/2}\text{Pr}^{1/3}}$ (Eq. 12). By setting the radius of laser beam as typical length scale $L = 0.5$ mm, the estimated thickness of thermal boundary layer is $\delta_{\text{th}} = 0.58$ mm at $T = 293$ K and $\delta_{\text{th}} = 0.33$ mm at $T = 373$ K. The thickness of TIL obtained from thermal images is about 0.3 mm (Supplementary Fig. 4d).

Supplementary Note 7. Model for bubble bouncing: R_{up}, R_{low} and f

The relevant forces. As shown in the sketch in the main text (Fig. 3), the downward Marangoni force (F_{m}^{-}), the upward Marangoni force (F_{m}^{+}), the buoyancy force (F_{b}), and the viscous force

(F_v) can be expressed as following in Ref. (*4*).

The downward Marangoni force is,

$$F_\mathrm{m}^- = \Delta\gamma \cdot R = \frac{\mathrm{d}\gamma}{\mathrm{d}T}\frac{\mathrm{d}T^-}{\mathrm{d}z} \cdot \min(2R, \delta_\mathrm{inv}) \cdot \pi R. \tag{17}$$

The buoyancy force is,

$$F_\mathrm{b} = \rho g \cdot \frac{4}{3}\pi R^3. \tag{18}$$

The viscous force according to Ref. (*5*) is,

$$F_\mathrm{v} = -12\pi\mu R v. \tag{19}$$

The upward Marangoni force depends on the position and motion status of the bubble. When bubble rests near the cover, the upward Marangoni force is,

$$F_\mathrm{m}^+ = \Delta\gamma \cdot R = \frac{\mathrm{d}\gamma}{\mathrm{d}T}\frac{\mathrm{d}T^+}{\mathrm{d}z} \cdot \max(0, 2R - \delta_\mathrm{inv}) \cdot \pi R. \tag{20}$$

When bubble moves in liquid, the upward Marangoni force is,

$$F_\mathrm{m}^+ = \Delta\gamma \cdot R = \frac{\mathrm{d}\gamma}{\mathrm{d}T}\frac{\mathrm{d}T^+}{\mathrm{d}z} \cdot 2\pi R^2. \tag{21}$$

When bubble rests near the cover, the temperature gradient $\mathrm{d}T/\mathrm{d}z$ in the expression of F_m^- (about 50 K/mm in experiments) is at least one order larger than it in F_m^+ (about 1 K/mm), thus in the analysis at the beginning of bouncing motion, the upward Marangoni force F_m^+ can be ignored. The viscous force is much smaller than other forces during the bouncing process and can be neglected here; but is important in the analysis for the horizontal motion and will be considered carefully.

Based on these expression, the total forces for the bubble motion can be obtained in Ref. (*6*),

$$F(t) = F_\mathrm{v} - \frac{4}{3}\pi\rho R(t)^3\ddot{z}(t) - \frac{2}{3}\pi\rho\left(\frac{\mathrm{d}\left[R(t)^3\dot{z}(t)\right]}{\mathrm{d}t} + 2R(t)^3\ddot{z}(t)\right) - F_\mathrm{b} - \left|F_\mathrm{m}^+\right|, \tag{22}$$

where the velocity of bubble motion $v = \dot{z}(t)$. Here the radius change during one bounce cycle is ignored, *i.e.,* $R(t) = \text{const}$.

Upper bound radius (R_{up}). For the upper bound radius (R_{up}) for bubble bouncing, as shown in Fig. 3b, the buoyancy force F_{b} is larger than upward Marangoni force F_{m}^{+} when bubble radius is large. By balancing the downward Marangoni force $F_{\text{m}}^{-} = \frac{\mathrm{d}\gamma}{\mathrm{d}T}\frac{\mathrm{d}T^{-}}{\mathrm{d}z} \cdot \delta_{\text{inv}} \cdot \pi R$ with the buoyancy force $F_{\text{b}} = \rho g \cdot \frac{4}{3}\pi R^3$, the upper bound radius for bubble bouncing is,

$$R_{\text{up}}^2 = \frac{3}{4\rho g}\frac{\mathrm{d}\gamma}{\mathrm{d}T}\frac{\mathrm{d}T^{-}}{\mathrm{d}z}\delta_{\text{inv}}, \tag{23}$$

where the temperature gradient of surface tension for water $|\mathrm{d}\gamma/\mathrm{d}T| = 2.02 \times 10^{-4}$ kg/(s^2 · K), the elevated temperature $\Delta T \propto P$ according to Eq. 4 and Eq. 8, and the thickness of TIL $\delta_{\text{inv}} \propto P^{-1/4}$ according to Eq. 16. Thus, the scaling for upper bound radius $R_{\text{up}} \propto P^{-3/8}$.

In experiments, the maximum of temperature gradient $\mathrm{d}T/\mathrm{d}z$ is about 100 K/mm for laser power $P = 15$ W, and the thickness of TIL is about 0.3 mm. Thus, the corresponding estimated upper bound radius $R_{\text{up}} \approx 0.69$ mm is comparable with the experimental value of $R_{\text{up}} = 0.75 \pm 0.07$ mm in Fig. 3e.

Lower bound radius (R_{low}). For the lower bound radius (R_{low}) for bubble bouncing, as shown in Fig. 3b, the upward Marangoni force F_{m}^{+} is larger than the buoyancy force F_{b} when bubble radius is small, thus in this analysis we consider the upward Marangoni force is dominant. As shown in Fig. 1c, we note that the bouncing begins with a small amplitude, to identify the lower threshold of bubble bouncing, a criterion for the bubble vertical displacement H_{t} is needed. Thus, to eliminate the effect of fluctuation of bubble radius and lateral oscillation of bubble, we set the lower bouncing threshold of $H_{\text{t,cr}} \approx 0.1R_{\text{low}}$ that only when the bubble vertical displacement is comparable with 0.1 of its radius, the bubble is bouncing. To obtain the bubble vertical displacement H_{t}, consider the energy conservation in Ref. (*6*), the work done by downward Marangoni force (F_{m}^{-}) within TIL,

$$W_{\mathrm{m}}^{-}=\zeta\frac{\mathrm{d}\gamma}{\mathrm{d}T}\frac{\mathrm{d}T^{-}}{\mathrm{d}z}\cdot 2\pi R^{2}\delta_{\mathrm{inv}}, \tag{24}$$

where $\zeta\,(0<\zeta<1)$ is the ratio between the work done by F_{m}^{-} and the surface energy of bubble.

The work done by upward Marangoni force (F_{m}^{+}) when bubble reaches H_{t},

$$W_{\mathrm{m}}^{+}=F_{\mathrm{m}}^{+}\cdot H_{\mathrm{t}}=\frac{\mathrm{d}\gamma}{\mathrm{d}T}\frac{\mathrm{d}T^{+}}{\mathrm{d}z}\cdot 2\pi R^{2}\cdot H_{\mathrm{t}}. \tag{25}$$

Thus, by equating $W_{\mathrm{m}}^{-}=W_{\mathrm{m}}^{+}$, the lower bound radius R_{low},

$$R_{\mathrm{low}}\approx 10H_{\mathrm{t,cr}}=\frac{\mathrm{d}T^{-}/\mathrm{d}z}{\mathrm{d}T^{+}/\mathrm{d}z}\cdot 10\zeta\delta_{\mathrm{inv}}. \tag{26}$$

Although the detail expression of temperature gradient $\mathrm{d}T/\mathrm{d}z$ is different, however the scaling relation with laser power is same, according to Eq. 4, Eq. 8 and Eq. 13. Thus, the scaling relation for the lower bound radius $R_{\mathrm{low}}\propto H_{\mathrm{t,cr}}\propto\delta_{\mathrm{inv}}\propto P^{-1/4}$ according to Eq. 16.

In experiments, the temperature distribution at the beginning of bubble bounce is shown in Fig. 2b. The temperature gradient within TIL $\mathrm{d}T^{-}/\mathrm{d}z\approx 15$ K/mm, and the temperature gradient outside TIL $\mathrm{d}T^{+}/\mathrm{d}z\approx 1$ K/mm. Thus, by setting $\zeta=0.01$, the corresponding estimated lower bound radius $R_{\mathrm{low}}\approx 0.45$ mm; the lower bound radius for bubble bounce in experiments $R_{\mathrm{low}}=0.37\pm 0.07$ mm in Fig. 3e, comparable with the theoretical estimation.

Bouncing frequency. For the frequency of bubble bouncing, according to the analysis of forces applied on bubble (Eq. 22), by ignoring the viscous force and radius change, the equation is simplified as below,

$$\frac{10}{3}\pi\rho R^{3}\ddot{z}+F_{\mathrm{b}}+|F_{\mathrm{m}}^{+}|=0. \tag{27}$$

where the first term reflects added mass from the liquid acceleration.

The initial velocity of bubble motion ($v_{\mathrm{b},0}$) is determined by energy conservation in Ref. (*6*). The work done by downward Marangoni force (F_{m}^{-}) within TIL is,

$$W_{\mathrm{m}}^{-} = \zeta \frac{\mathrm{d}\gamma}{\mathrm{d}T} \frac{\mathrm{d}T^{-}}{\mathrm{d}z} \cdot 2\pi R^2 \delta_{\mathrm{inv}}, \tag{28}$$

where $\zeta\,(0 < \zeta < 1)$ is the ratio between the work done by F_{m}^{-} and the surface energy of bubble.

This energy is converted into the kinetic energy $E_{\mathrm{k}} = 10\pi\rho R^3 v_{\mathrm{b},0}^2/3$. From $W_{\mathrm{m}}^{-} = E_{\mathrm{k}}$, the initial velocity of bubble is,

$$v_{\mathrm{b},0} = \sqrt{\frac{3}{5\rho}\left|\frac{\mathrm{d}\gamma}{\mathrm{d}T}\frac{\mathrm{d}T^{-}}{\mathrm{d}z}\right|\frac{\zeta\delta_{\mathrm{inv}}}{R}}. \tag{29}$$

Then,the frequency of bubble bouncing f can be obtained,

$$f = \frac{\ddot{z}}{2v_{\mathrm{b},0}}. \tag{30}$$

For small bubble, the upward Marangoni force F_{m}^{+} is dominant,

$$f = \frac{|F_{\mathrm{m}}^{+}|}{2v_{\mathrm{b},0} \cdot 10\pi\rho R^3/3} = \sqrt{\frac{3|\mathrm{d}\gamma/\mathrm{d}T|}{20\rho\zeta R\delta_{\mathrm{inv}}|\mathrm{d}T^{-}/\mathrm{d}z|}} \cdot \left|\frac{\mathrm{d}T^{+}}{\mathrm{d}z}\right| = C_1 R^{-1/2}. \tag{31}$$

In experiments, the average temperature gradient within TIL $\mathrm{d}T^{-}/\mathrm{d}z \approx 50$ K/mm, and the average temperature gradient outsider TIL $\mathrm{d}T^{+}/\mathrm{d}z \approx 1$ K/mm, and $\zeta = 0.01$ is assumed to be the same as the analysis for R_{low}. Thus, the corresponding estimated prefactor in the scaling relation $C_{\mathrm{m}} \approx 14.1$, which is comparable with the experimental fitted prefactor $C_1 = 16$ (Fig. 3f).

For large bubble, the buoyancy force F_{b} is dominant,

$$f = \frac{F_{\mathrm{b}}}{2v_{\mathrm{b},0} \cdot 10\pi\rho R^3/3} = \sqrt{\frac{\rho g^2 R}{15\left|\frac{\mathrm{d}\gamma}{\mathrm{d}T}\right|\left|\frac{\mathrm{d}T^{-}}{\mathrm{d}z}\right|\zeta\delta_{\mathrm{inv}}}} = C_2 R^{1/2}. \tag{32}$$

We compared the estimated frequency with the observed frequency of bubble bouncing. The initial velocity of bubble motion $v_{\mathrm{b},0} \approx 0.1$ m/s according to the snapshot shown in Fig. 1a, and for large bubble ($R = 0.9$ mm), the corresponding estimated frequency of bubble bouncing $f = \frac{F_\mathrm{b}}{2v_{\mathrm{b},0} \cdot 10\pi\rho R^3/3} = 19.6$ Hz. The observed frequency for $R = 0.9$ mm is $f = 19.2 \pm 2.17$ Hz (Fig. 3f), consisitent with the theoretical estimation.

Thus, for a given laser power P, the scaling relation for the frequency of bubble bouncing is $f \propto R^{-1/2}$ for a small bubble, and $f \propto R^{1/2}$ for a large bubble. This scaling relationship has a remarkable agreement with experiments.

Supplementary Note 8. Model for the dancing bubble: R_up and v_cr

When the laser spot is translating horizontally, we need to consider the transient temperature evolution along both r and z direction. A local dwell timescale (τ_d) is introduced as a typical laser heating timescale during laser translating, $\tau_\mathrm{d} = 2r_\mathrm{l}/v_\mathrm{l}$, which is related with laser beam size r_l and translation speed v_l. According to the temperature profile in Supplementary Fig. 4, we define a critical transition point τ_c: for $\tau < \tau_\mathrm{c}$, the elevated temperature ΔT increases linearly with τ; while for $\tau > \tau_\mathrm{c}$, ΔT reaches a steady state approximately.

Upper bound radius. Similar to the analysis of upper bound radius for bouncing, by balancing the downward Marangoni force ($F_\mathrm{m}^- = \frac{\mathrm{d}\gamma}{\mathrm{d}T}\frac{\mathrm{d}T}{\mathrm{d}z} \cdot \delta_\mathrm{inv} \cdot \pi R$) with buoyancy force ($F_\mathrm{b} = \rho g \cdot \frac{4}{3}\pi R^3$) (the temperature gradient $\frac{\mathrm{d}T}{\mathrm{d}z} \propto P \cdot \min(\tau_\mathrm{d}, \tau_\mathrm{c})$ according to Eq. 4), the upper bound radius (R_up) for bubble dancing can be obtained,

$$R_\mathrm{up}^2 = \frac{3\delta_\mathrm{inv}}{4\rho g}\frac{\mathrm{d}\gamma}{\mathrm{d}T}\frac{\mathrm{d}T}{\mathrm{d}z} \propto \delta_\mathrm{inv}\Delta T \propto P^{3/4}\min(\tau_\mathrm{d}, \tau_\mathrm{c}) \tag{33}$$

In experiments, the critical timescale for heat transfer (as shown in Supplementary Fig. 4b) is $\tau_\mathrm{c} \approx 1$ s, then the critical translating velocity of laser spot $v_\mathrm{l,cr} = 2r_\mathrm{l}/\tau_\mathrm{c} \approx 1$ mm/s ($\tau_\mathrm{c} = \tau_\mathrm{d}$). And the intersection of the two lines (Fig. 4c) in experiments is $v_\mathrm{l,cr} \approx 1.3$ mm/s, which is

consistent with the theoretical estimation.

In the limit of slow translating velocity, the maximum of temperature gradient $\mathrm{d}T/\mathrm{d}z$ is about 100 K/mm for laser power $P = 15$ W in experiments. Thus, the corresponding estimated value $P^{3/4}/R_{\mathrm{up}}^2 \approx 16.0$ W$^{3/4}$/mm^2 again is comparable with the experimental value of $P^{3/4}/R_{\mathrm{up}}^2 = 10.5$ W$^{3/4}$/mm^2 (Fig. 4c).

Critical translation velocity. As discussed in the main text, consider the relationship between the center position of bubble (x_{b}) and the center position of laser (x_{l}). When bubble translates with the laser spot, the distance between bubble and laser remains constant $\Delta l = x_{\mathrm{l}} - x_{\mathrm{b}} =$ const, and the translation velocity $v = \dot{x}_{\mathrm{l}} = \dot{x}_{\mathrm{b}}$. Due to the zero adhesion of floating bubble, the translation motion velocity is determined by balancing the thermal Marangoni force $F_{\mathrm{m}} = \gamma_{\mathrm{th}}^{x} \cdot 2\pi r_{\mathrm{b}}^2 = \frac{\mathrm{d}\gamma}{\mathrm{d}T} \cdot \frac{\mathrm{d}T}{\mathrm{d}x} \cdot 2\pi r_{\mathrm{b}}^2$ with the Stokes drag force $F_{\mathrm{v}} = -12\pi\mu r_{\mathrm{b}} v$ in Ref. (*4*).

To estimate the temperature gradient induced by moving laser heating effect, the typical heating period is taken as $\tau \sim \Delta l/v$ for the time delay when laser and bubble arrive the same position. The laser intensity distribution is taken as gaussian distribution, and the temperature distribution along the horizontal (x) direction in Ref. (*1*),

$$T = T_0 + \frac{\alpha\eta(z)P}{\pi\rho c_{\mathrm{p}} r_{\mathrm{l}}^2} e^{-x^2/r_{\mathrm{l}}^2}\tau, \tag{34}$$

where $r_{\mathrm{b}}, r_{\mathrm{l}}$ are radius of bubble and laser spot, respectively, α is absorption coefficient, and $\eta(z)$ is penetration coefficient. Then the thermal Marangoni force can be derived (let $x = \Delta l$),

$$F_{\mathrm{m}} = \frac{\mathrm{d}\gamma}{\mathrm{d}T} \cdot 2\pi r_{\mathrm{b}}^2 \cdot \frac{\alpha\eta(z)P}{\pi\rho c_{\mathrm{p}} r_{\mathrm{l}}^2} e^{-\Delta l^2/r_{\mathrm{l}}^2} \cdot \frac{\Delta l}{v} \cdot \frac{-2\Delta l}{r_{\mathrm{l}}^2}. \tag{35}$$

By balancing the thermal Marangoni force and Stokes drag force, a critical translation velocity v_{cr} is obtained,

$$v_{\mathrm{cr}} = \sqrt{\left|\frac{\mathrm{d}\gamma}{\mathrm{d}T}\right| \cdot \frac{\alpha\eta(z)P}{3\pi\rho c_{\mathrm{p}}\mu} \cdot \frac{r_{\mathrm{b}}\Delta l^2}{r_{\mathrm{l}}^4} e^{-\Delta l^2/r_{\mathrm{l}}^2}}. \tag{36}$$

To estimate the critical translation velocity in our experiments, by setting $r_{\mathrm{b}} = r_{\mathrm{l}} = \Delta l =$0.5 mm, laser power $P =$20 W, and the absorption coefficient along z direction $\eta(z) = 0.5$, the critical translation velocity is $v_{\mathrm{cr}} \approx 4.1$ cm/s in theory. In the horizontal translation experiments, bubble tightly follows the laser spot as shown in Supplementary Fig. 7, even for the speed up to 40 mm/s, which is consistent with the theoretical estimation.

Supplementary Methods

1. Simulation for TIL

To validate the formation of TIL, we solve the Navier-Stokes equation and heat transfer equation in axisymmetric cylindrical coordinate (r, z) by considering the buoyancy force,

$$\rho \nabla \cdot \mathbf{u} = 0 \tag{37}$$

$$\rho \left(\frac{\partial \mathbf{u}}{\partial t} + \mathbf{u} \cdot \nabla \mathbf{u} \right) = -\nabla p + \mu \nabla^2 \mathbf{u} + \rho \mathbf{g} \tag{38}$$

$$\frac{\partial T}{\partial t} + \mathbf{u} \cdot \nabla T = \kappa \nabla^2 T + \dot{Q} \tag{39}$$

The blue region is water ($0 < r < r_{\mathrm{w}}, 0 < z < h_{\mathrm{w}}$), and the grey region is glass ($0 < r < r_{\mathrm{w}}, h_{\mathrm{w}} < z < h_{\mathrm{w}} + h_{\mathrm{s}}$). The initial temperature is room temperature ($T_0 = 20$°C) for both water and glass, and the initial velocity of water is zero. The boundary conditions are shown in sketch (Supplementary Fig. 8). No-slip boundary condition is applied at $z = 0, z = h_{\mathrm{w}}$ and $r = r_{\mathrm{w}}$. Adiabatic condition is applied at $z = 0, z = h_{\mathrm{w}} + h_{\mathrm{s}}$ and $r = r_{\mathrm{w}}$. Laser heating effect is introduced as a volumetric heat source with gaussian distribution in r direction and exponential distribution in z direction $\dot{Q}(r, z) \sim e^{-\alpha z - r^2/r_{\mathrm{l}}^2}$ according to Ref. (*1*). The density of water varies with temperature and the model is solved with COMSOL. Furthermore, five different top surface materials (air, PMMA, PDMS, quartz and sapphire) are considered in the simulation, whose properties are listed in Supplementary Tab. 1.

2. Simulation for bouncing bubble

To validate the mechanism for bouncing bubble induced by thermal Marangoni force which is produced in TIL, we decouple the complex relation between fluid flow and heat transfer by inputting temperature distribution based on experimental observation and former simulation for TIL, as shown in sketch (Supplementary Fig. 9). We solve the Navier-Stokes equation in

axisymmetric cylindrical coordinate (r, z), and use phase field method to track the liquid-gas interface.

The blue region is water ($0 < r < r_{\mathrm{w}}, 0 < z < h_{\mathrm{w}}$), and the white region is bubble ($r^2 + (z - h_{\mathrm{w}} + r_{\mathrm{b}})^2 < r_{\mathrm{b}}^2$). The given temperature distribution is based on experimental observation and former simulation. Negative temperature gradient ($\mathrm{d}T^-/\mathrm{d}z < 0$) in TIL ($h_{\mathrm{w}} - \delta_{\mathrm{inv}} < z < h_{\mathrm{w}}$), while positive temperature gradient ($\mathrm{d}T^+/\mathrm{d}z > 0$) outside TIL ($0 < z < h_{\mathrm{w}} - \delta_{\mathrm{inv}}$). No-slip boundary condition is applied at ($z = 0, z = h_{\mathrm{w}}$ and $r = r_{\mathrm{w}}$). The surface tension is input as a function of temperature $\gamma(T) = \gamma_0 + \frac{\mathrm{d}\gamma}{\mathrm{d}T}(T - T_0)$, and the model is solved with COMSOL.

Furthermore, according to the analysis in Fig. 3b in main text, four typical cases are simulated to validate our analysis, and setting parameters are listed in Supplementary Tab. 2.

Case I (Fig. 3g in main text) indicates that the upward Marangoni force (F_{m}^{+}) is comparable with buoyancy force (F_{b}), and both forces should be considered in the bouncing process. Case II (Supplementary Fig. 10a) indicates that the upward Marangoni force (F_{m}^{+}) is much larger than buoyancy force (F_{b}), thus the F_{b}^{+} is dominant while F_{b} is negligible. Case III (Supplementary Fig. 10b) indicates that F_{m}^{+} is much smaller than F_{b}, thus F_{b} is dominant while F_{m}^{+} is negligible.

Case IV (Supplementary Fig. 10c) is similar to Case III, except that TIL remains stable during bubble bouncing in Case IV, while TIL exists like a pulse ($0 < \tau < 5$ ms) in Case III. The bubble tends to stop at the equilibrium position in water bulk, which is different from the behaviors observed from experiments, indicating that TIL is not stable during bubble bouncing.

3. Simulation for dancing bubble

To confirm the dancing bubble behaviors induced by moving laser spot, the complex relation between fluid flow and heat transfer is decoupled by inputting temperature distribution similar to the former simulation for bouncing bubble (Supplementary Fig. 11). We solve the Navier-Stokes equation in Cartesian coordinate (x, y), using phase field method to track liquid-gas

interface.

The blue region is water ($0 < x < l_\mathrm{w}, 0 < y < h_\mathrm{w}$), and the white region is bubble ($(x - x_\mathrm{b})^2 + (y - h_\mathrm{w} + r_\mathrm{b})^2 < r_\mathrm{b}^2$). The temperature distribution is input based on experimental observation and former simulation. Negative temperature gradient ($\mathrm{d}T^-/\mathrm{d}y < 0$) in TIL ($h_\mathrm{w} - \delta_\mathrm{inv} < y < h_\mathrm{w}$), while a positive temperature gradient ($\mathrm{d}T^+/\mathrm{d}y > 0$) outside TIL ($0 < y < h_\mathrm{w} - \delta_\mathrm{inv}$). Here, to simplify the simulation model, the temperature gradient is set to be zero outside TIL, which is reasonable in the case that buoyancy force is dominant restoring force in the rebound process (Case III in simulation for bubble bouncing). Additionally, a positive temperature gradient ($\mathrm{d}T^+/\mathrm{d}x > 0$) is inputted along x direction in water bulk ($0 < x < l_\mathrm{w}, 0 < y < h_\mathrm{w}$). No-slip boundary condition is applied at ($y = 0, y = h_\mathrm{w}$ and $x = l_\mathrm{w}$). The surface tension is expressed as $\gamma(T) = \gamma_0 + \frac{\mathrm{d}\gamma}{\mathrm{d}T}(T - T_0)$, and the model is solved with COMSOL.

4. Simulation for bubble leaping over a wall

To demonstrate the potential application of dancing bubble, we introduce a wall or obstacle on the path of bubble movement. The complex relation between fluid flow and heat transfer is decoupled by inputting temperature distribution similar to the former simulation for dancing bubble, as shown in sketch (Supplementary Fig. 12). We solve the Navier-Stokes equation in Cartesian coordinate (x, y), using phase field method to track liquid-gas interface.

The blue region is water ($0 < x < l_\mathrm{w}, 0 < y < h_\mathrm{w}$), and the white region is bubble ($(x - x_\mathrm{b})^2 + (y - h_\mathrm{w} + r_\mathrm{b})^2 < r_\mathrm{b}^2$). The temperature distribution is input based on experimental observation and former simulation. Negative temperature gradient ($\mathrm{d}T^-/\mathrm{d}y < 0$) in TIL ($h_\mathrm{w} - \delta_\mathrm{inv} < y < h_\mathrm{w}$), while positive temperature gradient ($\mathrm{d}T^+/\mathrm{d}y > 0$) outside TIL ($0 < y < h_\mathrm{w} - \delta_\mathrm{inv}$). Here, to further simplify the simulation model, the temperature gradient is set to be zero outside TIL, which is reasonable in the case that buoyancy force is dominant restoring force in the rebound process (Case III in simulation for bubble bouncing). Additionally, positive

temperature gradient ($\mathrm{d}T^{+}/\mathrm{d}x > 0$) is input along x direction in water bulk ($0 < x < l_{\mathrm{w}}, 0 < y < h_{\mathrm{w}}$). No-slip boundary condition is applied at ($y = 0, y = h_{\mathrm{w}}$ and $x = l_{\mathrm{w}}$). The surface tension is input as a function of temperature $\gamma(T) = \gamma_0 + \frac{\mathrm{d}\gamma}{\mathrm{d}T}(T - T_0)$, and the model is solved with COMSOL.

When the negative temperature gradient equals to zero ($\mathrm{d}T^{-}/\mathrm{d}y = 0$), the bubble moves horizontally, thus it is trapped by the wall (Supplementary Fig. 13). When the negative temperature gradient is large enough, the bubble can leap over the wall (Fig. 5b in the main text).

Supplementary Figures

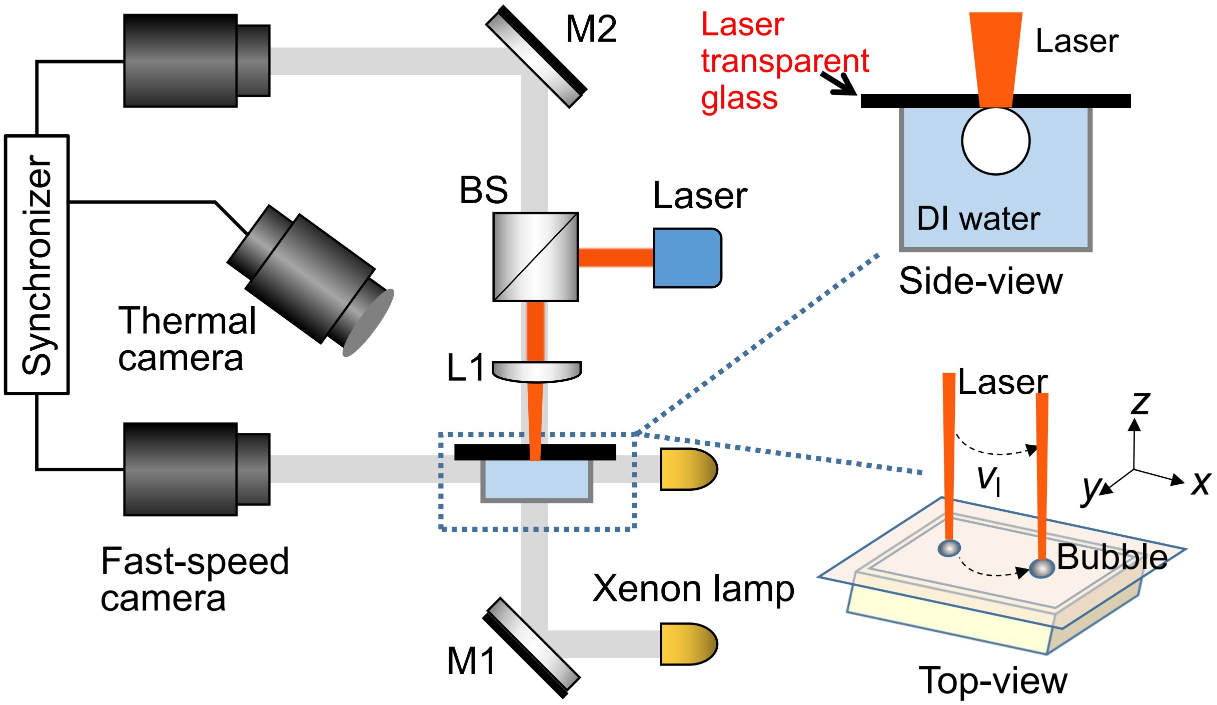

Supplementary Fig. 1: **Sketch for experimental setup.**

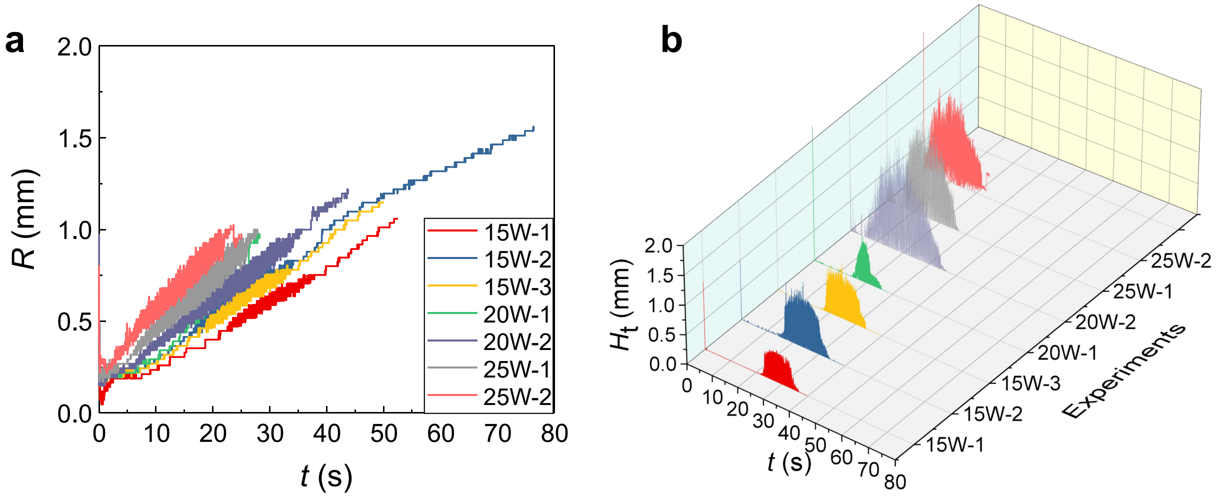

Supplementary Fig. 2: **Reproducibility of bouncing behavior for the bubble.** (**a**) The bubble radius R versus time t, and (**b**) H_{t} versus time t for three experiments at three different laser powers. Source data are provided as a Source Data file.

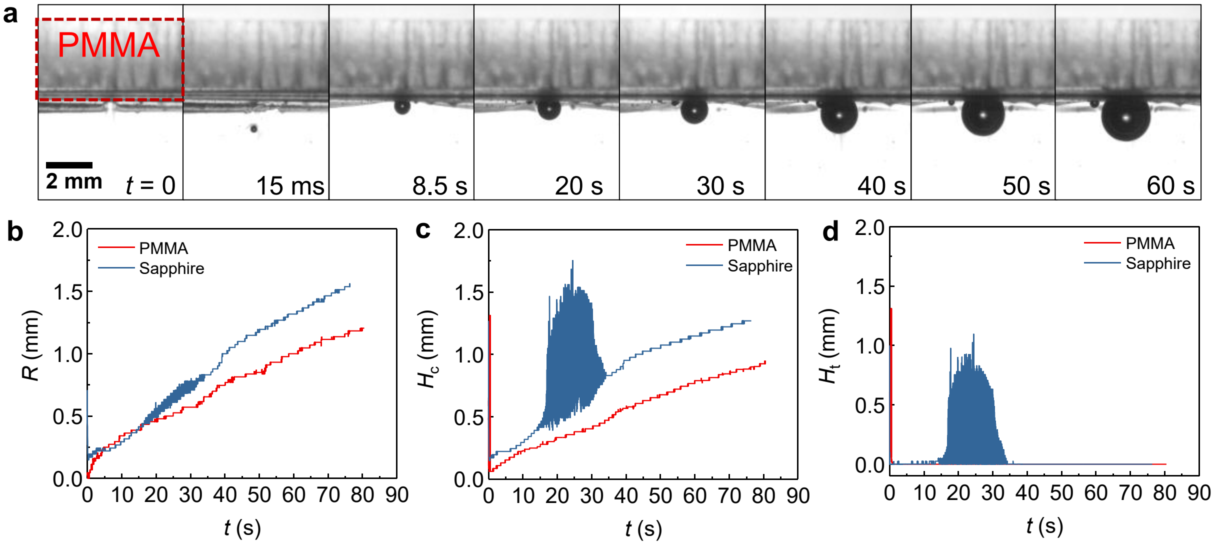

Supplementary Fig. 3: **Snapshots and comparison for experiments with different cover materials.** (**a**) High-speed images for the produced bubble within water for PMMA cover. (**b-d**) The bubble radius R, central position H_c, and topmost position H_t versus time t, respectively. Source data are provided as a Source Data file.

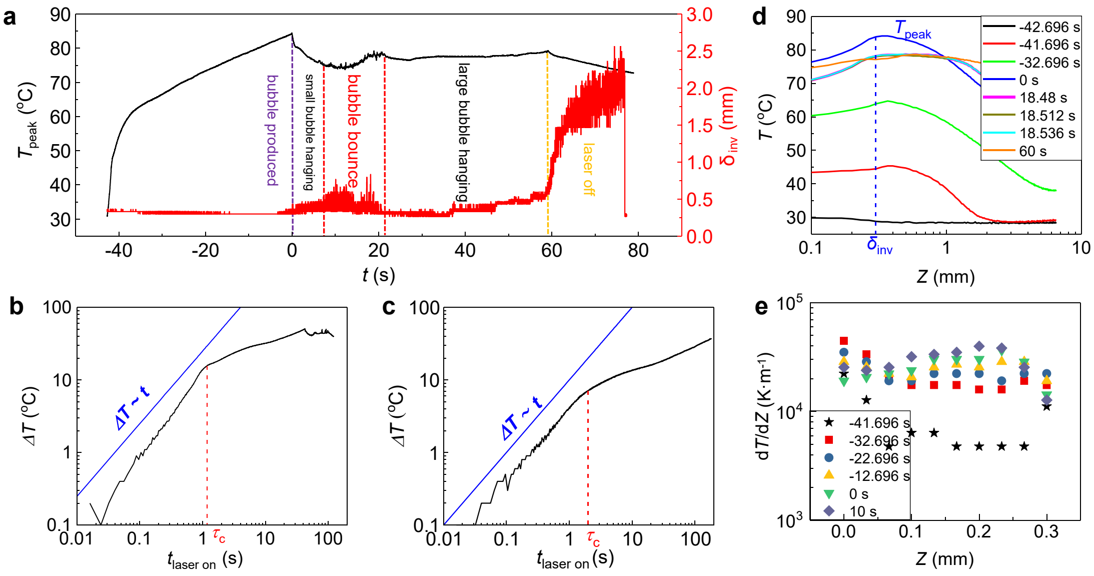

Supplementary Fig. 4: **Measured temperature during laser impacting on water.** (**a**) Evolution of peak temperature (T_{peak}) and the corresponding location δ_{inv} ($P = 15$ W with bubble formation) from the thermal images. (**b, c**) During the initial pre-heating stage, the evolution of elevated temperature (ΔT) for $P = 15$ W with and $P = 10$ W without bubble formation. (**d**) Temperature profile along the Z direction for $P = 15$ W with bubble formation. (**e**) The strong temperature gradient $\mathrm{d}T/\mathrm{d}Z$ reaches about 50 K/mm in TIL. Source data are provided as a Source Data file.

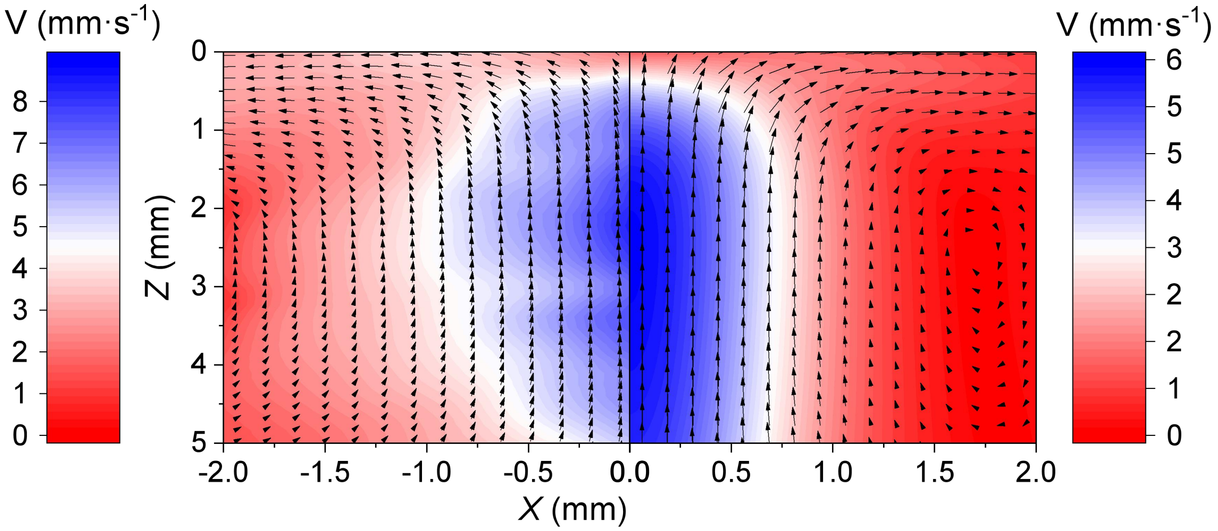

Supplementary Fig. 5: **Thermal buoyancy flow field.** Flows from PIV in experiment (left) is comparable with simulation (right), and the maximum velocity is about 10 mm/s. Source data are provided as a Source Data file.

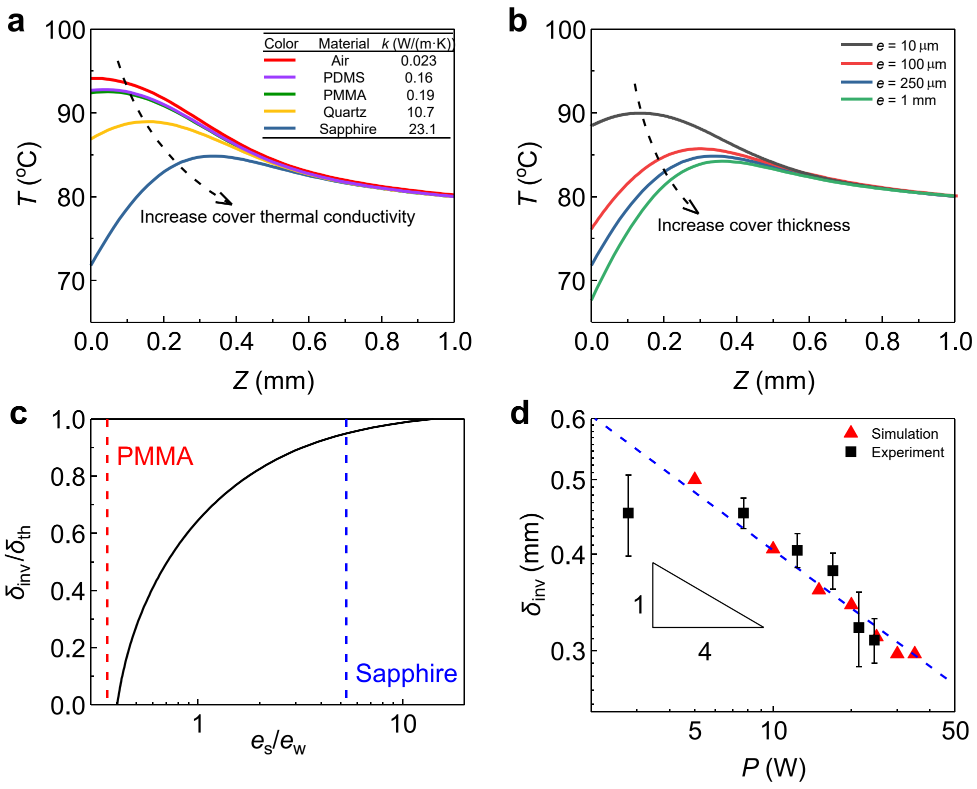

Supplementary Fig. 6: **Model for TIL.** (**a**) Temperature profile for different cover materials obtained from simulation. (**b**) Temperature profile for sapphire cover with different thickness obtained from simulation. (**c**) Dimensionless thickness of TIL $\delta_{\rm inv}$ dependence on the thermal effusivity of cover material. (**d**) The scaling relation between the thickness of TIL $\delta_{\rm inv}$ and laser power P in experiments and simulation. The error bars of the data in (**d** denote the standard deviation of at least three measurements. Source data are provided as a Source Data file.

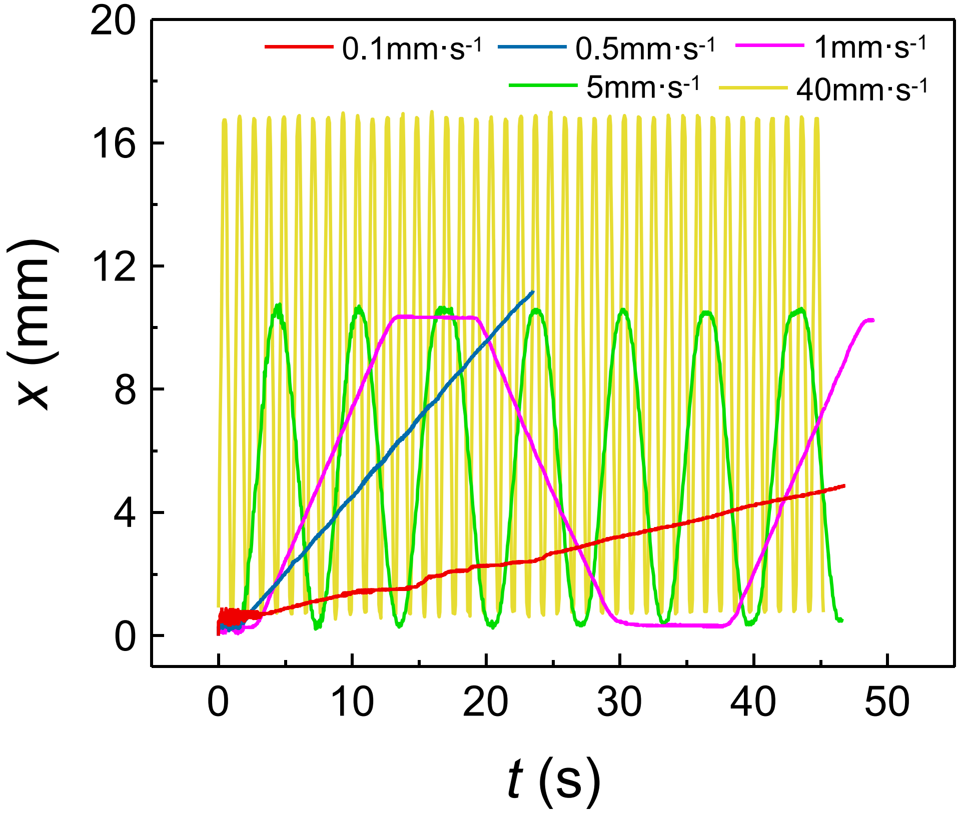

Supplementary Fig. 7: **Steerability of floating bubble.** Source data are provided as a Source Data file.

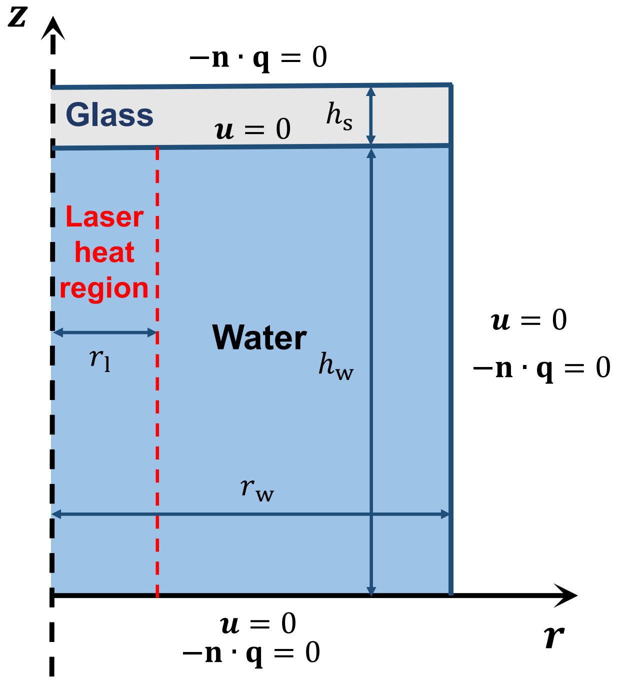

Supplementary Fig. 8: **Sketch for boundary conditions for TIL in simulation.**

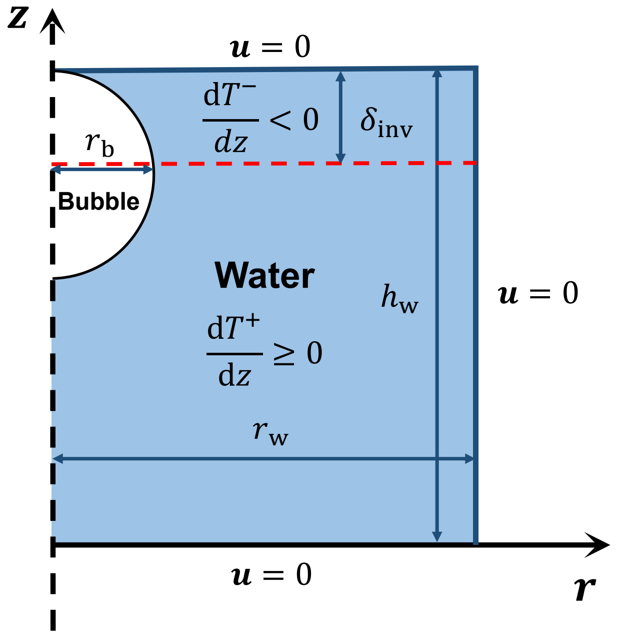

Supplementary Fig. 9: **Sketch for boundary conditions for bouncing bubble in the simulation.**

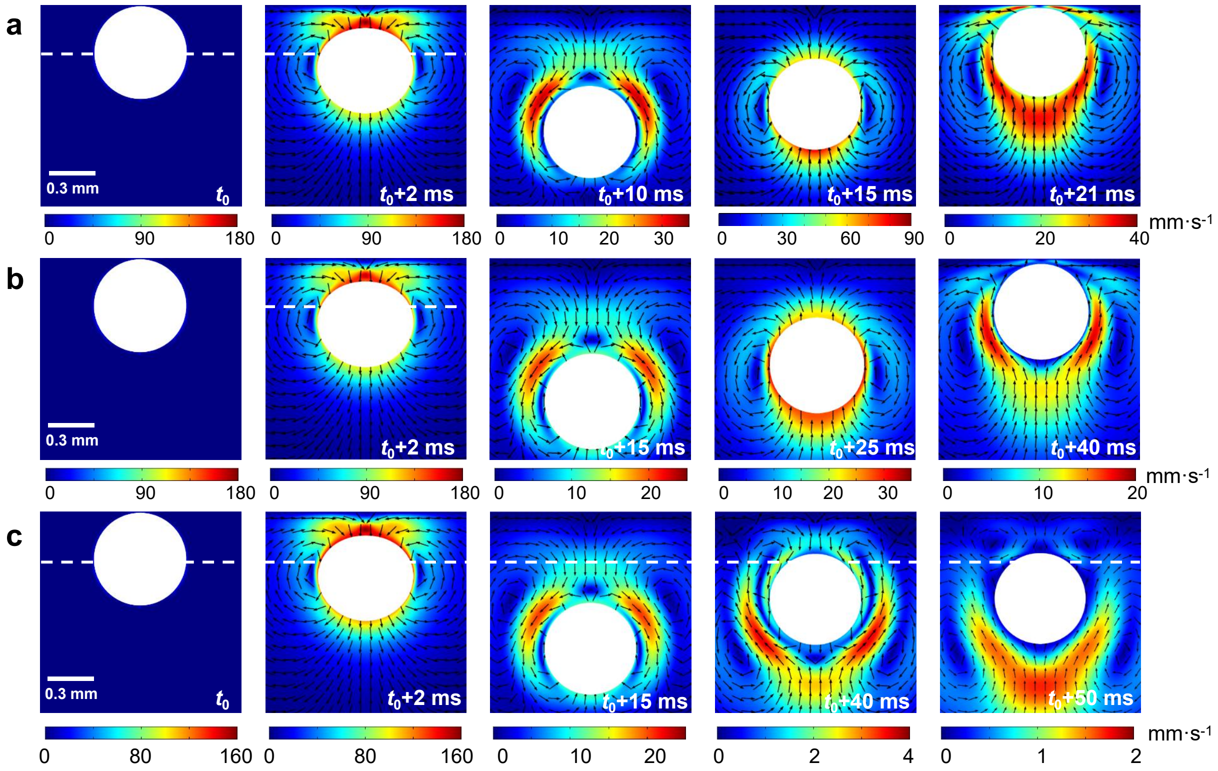

Supplementary Fig. 10: **Simulation for bubble bouncing.** (**a**) The upward Marangoni force (F_m^{+}) is dominant for Case II. (**b**) The buoyancy force (F_b) is dominant for Case III. (**c**) TIL remains stable during bubble bouncing for Case IV.

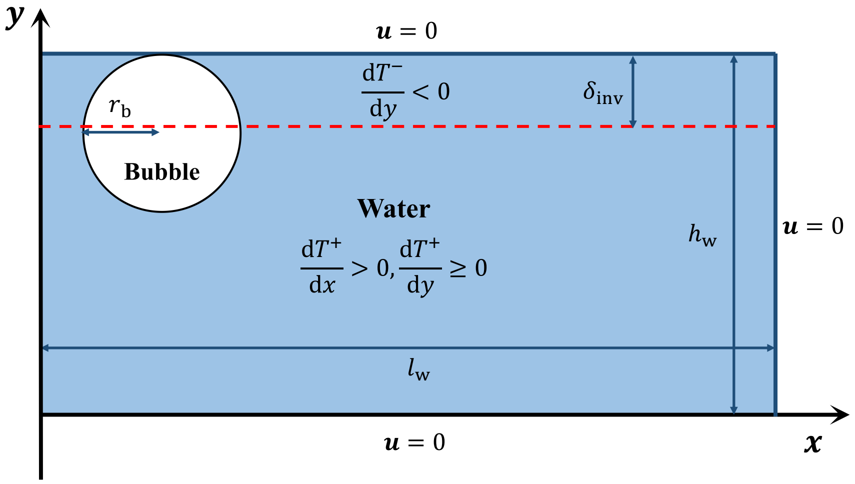

Supplementary Fig. 11: **Sketch for boundary conditions in the simulation for dancing bubble.**

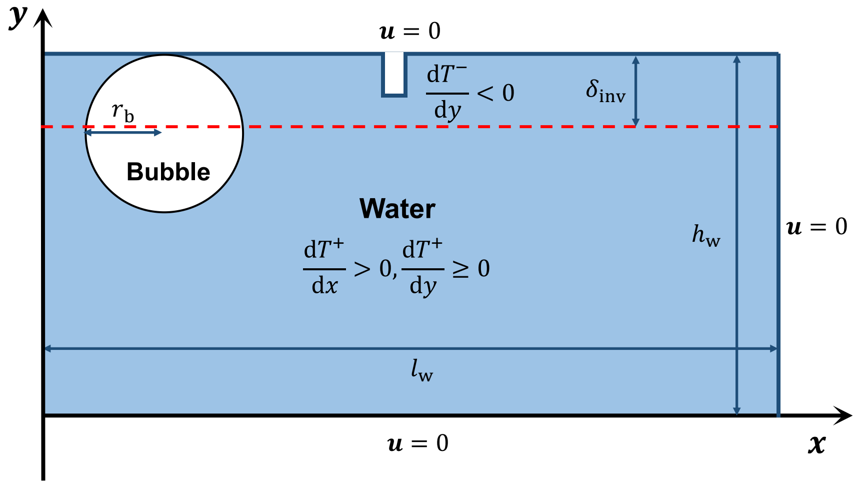

Supplementary Fig. 12: **Sketch for boundary conditions in the simulation for bubble leaping over a wall.**

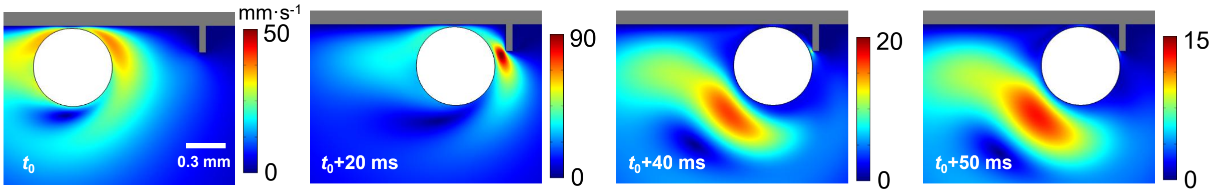

Supplementary Fig. 13: **Simulation for translating bubble trapped by the wall.**

Supplementary Tables

Supplementary Tab. 1: Material Properties ($T = 293.15$K)

Properties	Density	Thermal conductivity	Heat capacity	Thermal diffusivity	Thermal effusivity
Notation	ρ	k	c_{p}	κ	$e_{\mathrm{i}} = \sqrt{k_{\mathrm{i}}\rho_{\mathrm{i}}c_{\mathrm{pi}}}$
Units	kg/m^3	W/(m $\cdot$ K)	kJ/(kg $\cdot$K)	m^2/s	W$\sqrt{\mathrm{s}}$/m^{2}K
Water	1000	0.59	4.2	1.4×10^{-7}	1574.2
Sapphire	3980	23.1	0.761	7.63×10^{-6}	8364.5
Quartz	2200	1.32	0.772	7.77×10^{-7}	1497.3
PMMA	1190	0.19	1.42	1.12×10^{-7}	566.6
PDMS	970	0.16	1.46	1.13×10^{-7}	476.0

Supplementary Tab. 2: Simulation settings for four typical cases of bubble bouncing

	Negative gradient	Pulse	Positive gradient	Buoyancy force	Snapshots
Notation	$\lvert \mathrm{d}T^{-}/\mathrm{d}z \rvert$	τ	$\lvert \mathrm{d}T^{+}/\mathrm{d}z \rvert$	F_b	
Unit	K/mm	ms	K/mm	N	
Case I	50	$0\leq \tau \leq 5$	2.5	On	Fig. 3g
Case II	50	$0\leq \tau \leq 5$	10	Off	Supplementary Fig. 10a
Case III	50	$0\leq \tau \leq 5$	0	On	Supplementary Fig. 10b
Case IV	50	$0\leq \tau \leq 50$	0	On	Supplementary Fig. 10c

Supplementary Tab. 3: Definition of notation

Notation	Definition	Unit	Value
$\mathrm{d}\gamma/\mathrm{d}T$	Gradient of surface tension of water	$\mathrm{kg/(s^2 \cdot K)}$	-2× 10^{-4}
β	Thermal expansion coefficient of water	$\mathrm{1/K}$	2×10^{-4}
α	Attenuation coefficient of laser in water	$\mathrm{1/m}$	45
ρ	Density of water	$\mathrm{kg/m^3}$	998 (293 K), 958 (373 K)
c_p	Heat capacity of water	$\mathrm{kJ/(kg \cdot K)}$	4.18 (293 K), 4.22 (373 K)
k	Thermal conductivity of water	$\mathrm{W/(m \cdot K)}$	0.60 (293 K), 0.68 (373 K)
μ	Dynamic viscosity of water	$\mathrm{mPa \cdot s}$	1.00 (293 K), 0.28 (373 K)
Pr	Prandtl number of water	1	6.99 (293 K), 1.76 (373 K)

Supplementary Tab. 4: Comparison between theoretical estimation and experimental value

Notation	Definition	Theory	Experiments
V_{b}	Velocity of buoyancy flow	8.9 mm/s	10 mm/s
δ_{inv}	Thickness of temperature inversion layer	0.33 mm	0.3 mm
R_{up}	Upper bound radius for bubble bounce (P =15 W)	0.69 mm	0.75±0.07 mm
R_{low}	Lower bound radius for bubble bounce (P =15 W)	0.45 mm	0.37±0.07 mm
C_1	Prefactor in expression for frequency dominant by F_{m}^{+}	14.1 mm$^{1/2}$/s	16 mm$^{1/2}$/s
f	Frequency dominant by F_{b} (R =0.9 mm)	19.6 Hz	19.2±2.17 Hz
$v_{\text{l,cr}}$	Critical translating velocity of laser spot for bubble dancing	1 mm/s	1.3 mm/s
$P^{3/4}/R_{\text{up}}^{2}$	Criterion for bubble dancing ($v_{\text{l}} < v_{\text{l,cr}}$)	16.0 W$^{3/4}$/mm^2	10.5 W$^{3/4}$/mm^2

Supplementary References

1. Y. Xu, R. Wang, S. Ma, L. Zhou, Y. R. Shen, C. Tian, Theoretical analysis and simulation of pulsed laser heating at interface. *J. Appl. Phys.* **123**, 025301 (2018).

2. T. L. Bergman, F. P. Incropera, D. P. DeWitt, A. S. Lavine, *Fundamentals of Heat and Mass Transfer* (John Wiley & Sons, 2011).

3. H. Carslaw, J. Jaeger, *Conduction of Heat in Solids* (Oxford: Clarendon Press, 1959).

4. C. E. Brennen, *Cavitation and Bubble Dynamics* (Cambridge University Press, 2014).

5. J. Magnaudet, D. Legendre, The viscous drag force on a spherical bubble with a time-dependent radius. *Phys. Fluids* **10**, 550–554 (1998).

6. B. Zeng, K. L. Chong, Y. Wang, C. Diddens, X. Li, M. Detert, H. J. Zandvliet, D. Lohse, Periodic bouncing of a plasmonic bubble in a binary liquid by competing solutal and thermal marangoni forces. *Proc. Natl. Acad. Sci. U. S. A.* **118** (2021).
