# Supplementary material for: Near-infrared-laser-navigated dancing bubble within water via a thermally conductive interface
Source: Nat Commun. 2022 Sep 30;13:5749. doi: 10.1038/s41467-022-33424-4 (PMC9525293; doi:10.1038/s41467-022-33424-4)
Supplement: Supplementary file 3 — Description of Additional Supplementary Files [file 41467_2022_33424_MOESM3_ESM.pdf]

Description of Additional Supplementary Files

Supplementary Movie 1. Motions of bubble bouncing in pure water (laser power P=15 W).
Supplementary Movie 2. Bubble moves with bounce at a translation speed of 1 mm/s.
Supplementary Movie 3. Bubble moves with bounce at a translation speed of 3 mm/s.
Supplementary Movie 4. Bubble moves without bounce at a translation speed of 5 mm/s.
Supplementary Movie 5. Horizontal translation movement of the floating bubble guided by laser with a speed up to 40 mm/s.
Supplementary Movie 6. 3D manipulation of the dancing bubble.
Supplementary Movie 7. Simulation for the dancing bubble leaping over a wall.
Supplementary Movie 8. Application of bubble coalescence guided by laser.
Supplementary Movie 9. Application of bubble encapsulated by a hexane droplet.
Supplementary Movie 10. Application of particles collection and transportation by bubble.
